# Proteomic Analysis of the Venom of Jellyfishes *Rhopilema esculentum* and *Sanderia malayensis*

**DOI:** 10.3390/md18120655

**Published:** 2020-12-21

**Authors:** Thomas C. N. Leung, Zhe Qu, Wenyan Nong, Jerome H. L. Hui, Sai Ming Ngai

**Affiliations:** 1 State Key Laboratory of Agrobiotechnology, School of Life Sciences, The Chinese University of Hong Kong, Hong Kong, China; chunningtleung@cuhk.edu.hk; 2 Simon F.S. Li Marine Science Laboratory, State Key Laboratory of Agrobiotechnology, School of Life Sciences, The Chinese University of Hong Kong, Hong Kong, China; quzhe@cuhk.edu.hk (Z.Q.); nongwenyan@cuhk.edu.hk (W.N.)

**Keywords:** jellyfish, *Rhopilema esculentum*, Sanderia malayensis, proteome, venom, toxin

## Abstract

Venomics, the study of biological venoms, could potentially provide a new source of therapeutic compounds, yet information on the venoms from marine organisms, including cnidarians (sea anemones, corals, and jellyfish), is limited. This study identified the putative toxins of two species of jellyfish—edible jellyfish *Rhopilema esculentum* Kishinouye, 1891, also known as flame jellyfish, and Amuska jellyfish *Sanderia malayensis* Goette, 1886. Utilizing nano-flow liquid chromatography tandem mass spectrometry (nLC–MS/MS), 3000 proteins were identified from the nematocysts in each of the above two jellyfish species. Forty and fifty-one putative toxins were identified in *R. esculentum* and *S. malayensis*, respectively, which were further classified into eight toxin families according to their predicted functions. Amongst the identified putative toxins, hemostasis-impairing toxins and proteases were found to be the most dominant members (>60%). The present study demonstrates the first proteomes of nematocysts from two jellyfish species with economic and environmental importance, and expands the foundation and understanding of cnidarian toxins.

## 1. Introduction

Phylum Cnidaria Hatschek, 1888, is one of the most ancient phyla that can be traced back to Cambrian [1]. This phylum is divided into five classes: Anthozoa Ehrenberg, 1834 (corals and sea anemones), Cubozoa Werner, 1973 (box jellyfish), Hydrozoa Owen, 1843, Scyphozoa Götte, 1887 (true jellyfish), and Staurozoa Marques & Collins, 2004 (stalked jellyfish) [2]. Approximately 12,000 extant species are known from freshwater and marine habitats worldwide, from shallow coastal waters to the deep seas [3,4]. Cnidarians are characterized by the presence of nematocytes, which constitute an important specialized cell type that assists prey capture and predator deterrence. Each nematocyte houses a unique organelle called the nematocyst. This Golgi apparatus-derived organelle consists of a capsule containing an inverted tubule immersed in a mixture of venomous substances [5]. Upon mechanical or chemical stimulation, the tubule everts and injects a venomous mixture into the prey or predator. Jellyfish possess nematocytes mainly on the tentacles [6]. The composition of the venom inside the nematocyst varies between different jellyfish species and may be a mixture of proteinaceous and non-proteinaceous toxins with hemolytic, neurotoxic, cytotoxic, and dermonecrotic activities [6]. Depending on the composition of the venom, the symptoms of jellyfish envenomation vary from species to species, ranging from mild local symptoms such as pruritus, pain, and burning sensation to serious manifestations, including hypotension, cardiac failure, respiratory distress, pulmonary edema, and even death [6,7,8,9].

Despite the potential hazard to health, evidence is accumulating for jellyfish venoms as promising sources of therapeutic agents. For example, peptide fractions of *Chrysaora quinquecirrha* Desor, 1848, venom have shown anticancer activities in an Ehrlich ascites carcinoma (EAC) tumor mouse model [10]. Similarly, *Nemopilema nomurai* Kishinouye, 1922, crude venom also exhibits anticancer activities in the HepG2 liver cancer cell line and HepG2 xenograft mouse models [11]. Furthermore, the crude venom of *Pelagia noctiluca* Forsskal, 1775, and its peptide fractions not only demonstrate anticancer activities in several cancer cell lines, but also show anti-inflammatory activities [12]. The protein components of *P. noctiluca* venom also exhibit analgesic functions in mouse models, and it has also been suggested that *P. noctiluca* venom is a promising source of neuroprotective drugs due to its plasma antibutyrylcholinestrasic activities [13]. However, the individual components that exhibit the therapeutic functions are not well characterized because the compositions of jellyfish venoms are not well studied, considering that of the 7235 animal toxins and venom proteins recorded in the Tox-Prot database, only six are derived from jellyfish (as of October 2020 [14]). This knowledge gap greatly hinders the discovery of potential drug candidates in jellyfish venom.

Recently, our group reported high-quality de novo reference genomes and transcriptomes for the edible jellyfish *Rhopilema esculentum* and the Amuska jellyfish *Sanderia malayensis*, as well as their transcriptomes [15]. Herein, we extend this study to identify the putative toxins in these two jellyfish species at the protein level. In this work, the venoms were enriched by isolating the nematocytes using the density gradient centrifugation technique. The nematocyte proteomes were determined by nano-flow liquid chromatography tandem mass spectrometry (nLC–MS/MS) and the putative toxins were identified by subsequent bioinformatic analysis.

This study provides the first insights into the venom proteomes of *R. esculentum* and *S. malayensis,* not only facilitating the screening, isolation, and characterization of their novel therapeutic compounds, but also providing clues to the evolutionary and ecological role of these toxins.

## 2. Results

### 2.1. Transcriptome and Protein Database Construction

**Figure 1:**
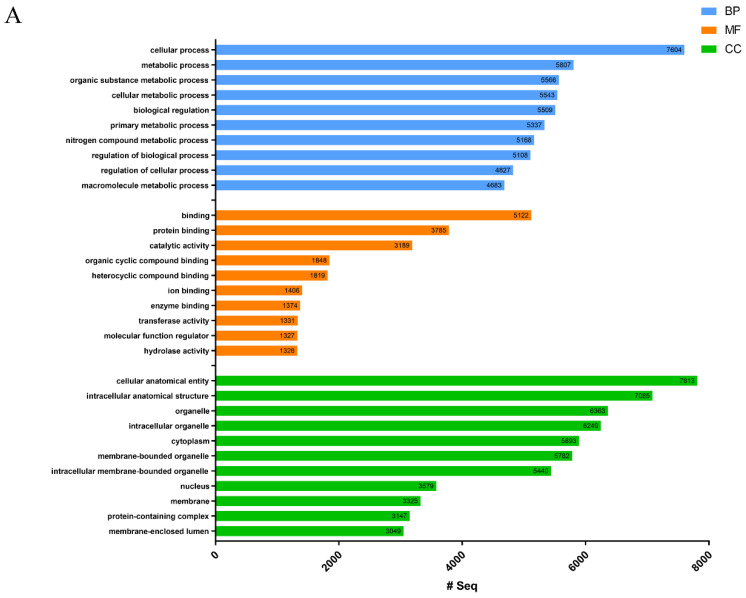

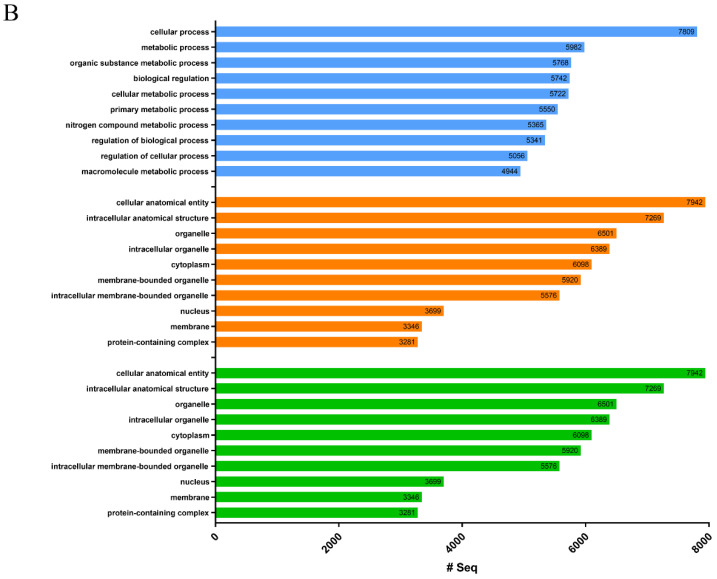
The 10 most represented Gene Oncology (GO) terms of the (**A**) *Rhopilema esculentum* and (**B**) *Sanderia malayensis* protein databases in the three domains of biological process (BP), molecular function (MF), and cellular component (CC) are presented.

**Figure 2:**
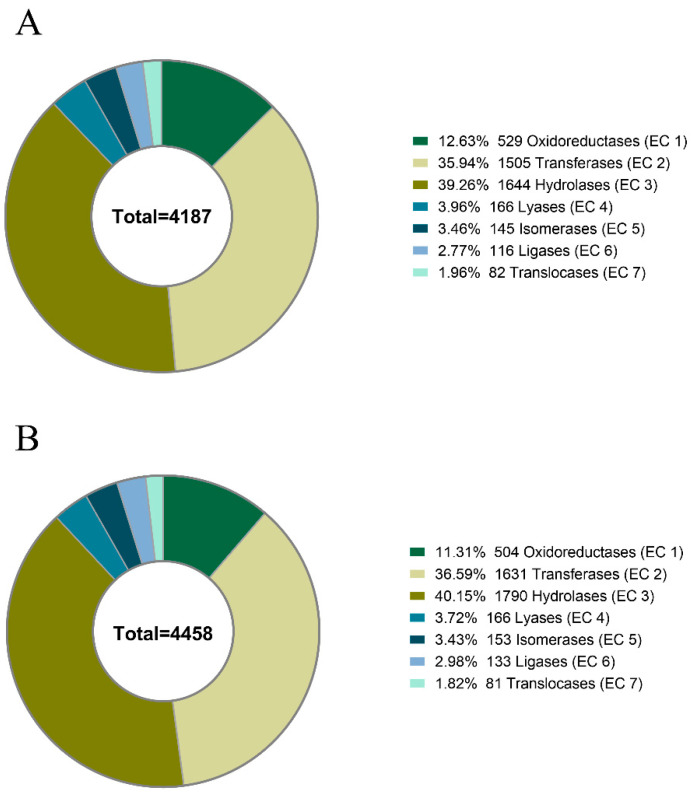
Distribution of the enzyme predicted by eggNOG-mapper in the protein databases of the (**A**) *R. esculentum* and (**B**) *S. malayensis* jellyfishes.

Next-generation sequencing (NGS) was used to construct the *R. esculentum* appendages and the *S. malayensis* tentacle transcriptome followed by gene model predictions using funannotate [15]. Based on the results of transcriptomic analysis, the *R. esculentum* and *S. malayensis* protein databases were generated with 18,923 and 26,914 protein sequences, respectively. Gene Ontology (GO) analysis was performed by the eggNOG-mapper [16] and annotations were assigned to three primary GO domains: biological process (BP), cellular component (CC), and molecular function (MF). In total, 8786 (46.43%) *R. esculentum* proteins and 9138 (33.95%) *S. malayensis* proteins were successfully annotated with 143,350 and 153,009 GO terms, respectively (Table 1 and Figure 1A,B). In addition, 4187 and 4485 enzymes were identified in *R. esculentum* and *S. malayensis*, respectively, classified according to their Enzyme Commission (EC) number. The proportional distributions of the enzymes in both species were similar, which were dominated by transferases and hydrolases (Table 1 and Figure 2). Furthermore, to identify and annotate the putative toxins, the protein sequences generated were run against the UniProt animal venom proteins and toxins database (Tox-Prot) using BLASTp [17]. Protein sequences with an e-value of <1.0 × 10^−5^ to entries in the database were used as input into “ToxClassifier” to exclude non-toxic homologs [18]. A total of 190 and 186 putative toxins were found in *R. esculentum* and *S. malayensis*, respectively. The toxin profiles of both species were similar in which the toxins could be classified into eight toxin families: hemostasis-impairing toxins, proteases, phospholipases, neurotoxins, cysteine-rich proteins, protease inhibitors, pore-forming toxins, and other toxins (Tables S1 and S2).

### 2.2. Identification of *R. esculentum* and *S. malayensis* Nematocyst Proteins by nano-LC-ESI MS/MS

**Figure 3:**
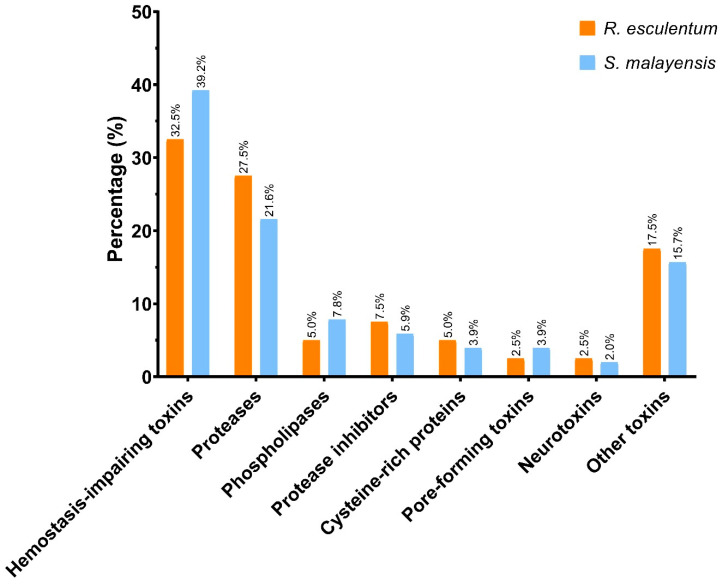
Graphical overview of the distribution of the toxin families identified in the *R. esculentum* and *S. malayensis* proteomes.

The nematocysts from *R. esculentum* and *S. malayensis* were purified and their protein profiles were revealed by proteomic analysis. A total of 3083 and 3559 proteins were identified in *R. esculentum* and *S. malayensis*, respectively (Tables S3 and S4), including 40 *R. esculentum* and 51 *S. malayensis* putative toxins. According to their predicted biological function, these toxins were classified into the eight toxin families (Table 2, Tables S5 and S6). The proportional distributions of these toxin families were also similar between the two species (Figure 3). Hemostasis-impairing toxins comprised the most abundant class of identified toxins, representing 32.5% and 39.2% of the *R. esculentum* and *S. malayensis* toxins, respectively, most of which were homologous to ryncolin, a family of proteinaceous toxins originally described from *Cerberus rynchops*. This family also includes a variety of C-type lectins (i.e., C-type lectin, C-type lectin lectoxin-Lio2, and galactose-specific lectin nattectin). In addition, other toxins such as prothrombin activator, coagulation factor V, coagulation factor X, and snaclec bothrojaracin subunit homologs were also identified in the proteome of both species.

Proteases comprised the second most predominant toxin family in both *R. esculentum* and *S. malayensis* proteome, accounting for 27.5% and 21.5% of the *R. esculentum* and *S. malayensis* putative toxins, respectively. Among this family, metalloproteinases were the most abundant. In the *R. esculentum* toxin proteome, three out of the seven metalloproteinases found were homologous to zinc metalloproteinase-disintegrin proteins. Meanwhile, a further two were neprilysin-1 homologs, another two were homologs of astacin-like metalloproteases. In the *S. malayensis* toxins, nine metalloproteinases were found, four of which were zinc metalloproteinase-disintegrin proteins. Four astacin-like metalloproteases and one neprilysin-1 were also detected.

Besides these two major classes of toxins, the *R. esculentum* and *S. malayensis* venoms also exhibited similar proportional distributions of other toxins. Meanwhile, l-amino-acid oxidase, acetylcholinesterase, and venom acid phosphatase were only found in *S. malayensis* venom, and U-actitoxin-Avd3j and calglandulin were only detected in *R. esculentum* venom.

### 2.3. Functional Analysis of the Putative Toxins

**Figure 4:**
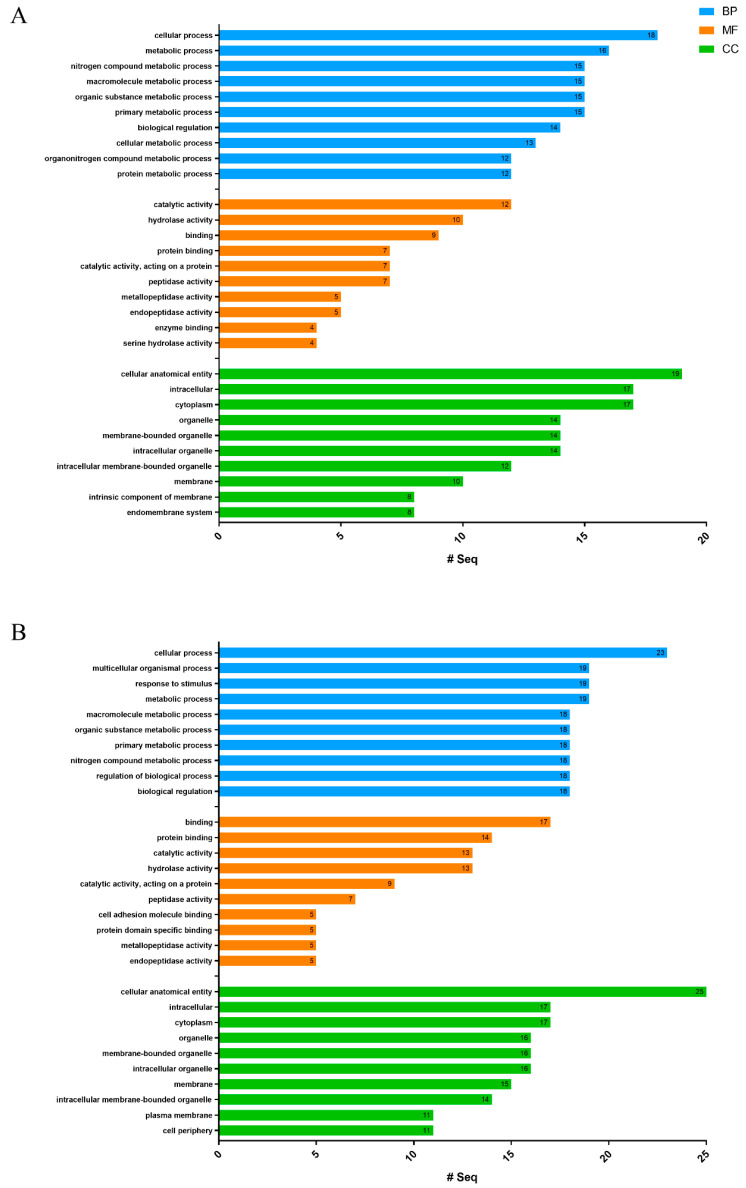
The 10 most represented GO terms of the (**A**) *R. esculentum* and (**B**) *S. malayensis* potential toxins in the three domains of biological process (BP), molecular function (MF), and cellular component (CC) are presented.

**Figure 5:**
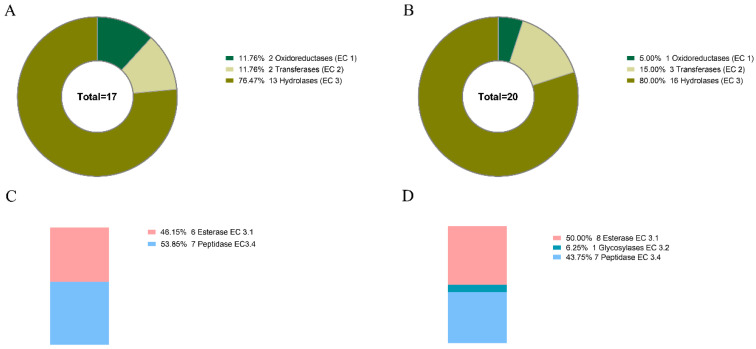
Distribution of the enzymes predicted by eggNOG-mapper in the putative toxins of (**A**) *R. esculentum* and (**B**) *S. malayensis* and the proportional distribution of the hydrolase subclass in (**C**) *R. esculentum* and (**D**) *S. malayensis*.

A total of 282 and 408 GO terms were assigned to 20 (50%) *R. esculentum* and 27 (54.9%) *S. malayensis* putative toxins, respectively (Tables S3 and S4). The 10 most represented GO terms in the three domains of biological process (BP), cellular component (CC), and molecular function (MF) are shown in Figure 4. Furthermore, the presence of signal peptides was predicted by SignalP, showing that 52.5% and 29.4% of the *R. esculentum* and *S. malayensis* putative toxins contain secretory signal peptides, respectively. Moreover, DeepLoc analysis indicated that 52.5% and 54.9% of the putative toxins were located in the extracellular region. Taken together, there 75% of the putative toxins in *R. esculentum* and 62.7% in *S. malayensis* were predicted as extracellular proteins (by SignalP and/or DeepLoc). Additionally, 16 and 20 enzymes were identified in the *R. esculentum* and *S. malayensis* putative toxins. In both species, hydrolase (EC 3) was the predominant enzyme (13 and 16 of *R. esculentum* and *S. malayensis* putative toxins, respectively; Figure 5A,C), the majority of which was comprised by esterase (EC3.1) and peptidase (EC 3.4) (Figure 5B,D).

### 2.4. InterProScan Analysis

**Figure 6:**
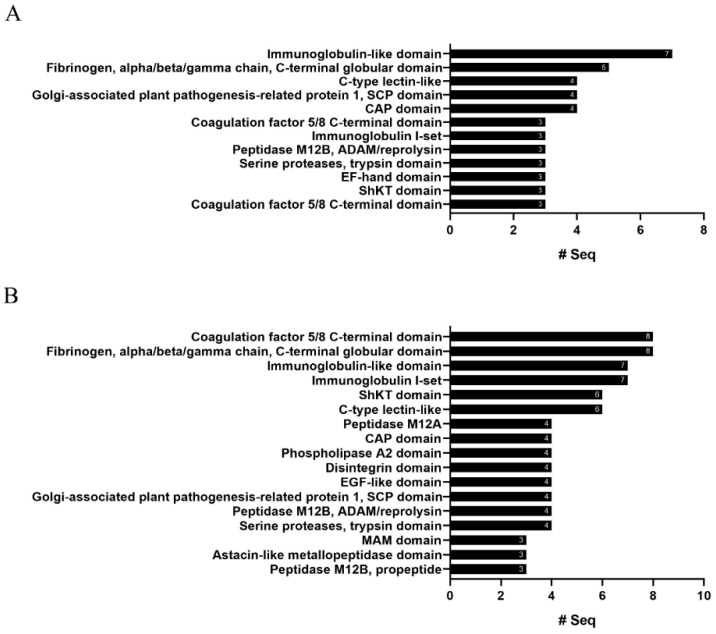
The highly represented protein domains of the (**A**) *R. esculentum* and (**B**) *S. malayensis* putative toxins. The number of proteins that contain the protein domain is labeled at the end of the column.

**Figure 7:**
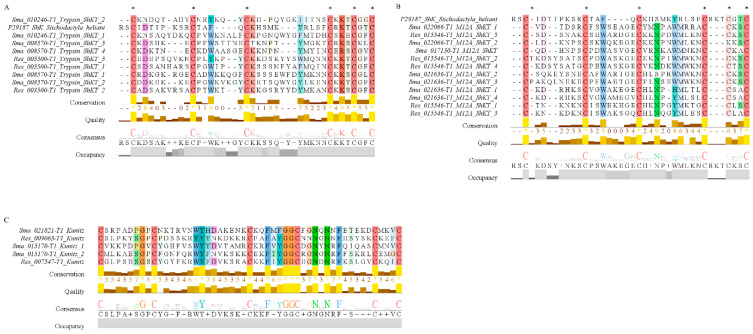
(**A**) Multiple sequence alignment of the ShKT domains from the trypsin toxins and the full length of Kappa-stichotoxin-She3a (ShK) from Sun anemone *Stichodactyla helianthus* (UniProt accession: P29187). (**B**) Multiple sequence alignment of the ShKT domains from the metalloproteases toxins and the full length of ShK (UniProt accession: P29187). (**C**) Multiple sequence alignment of the Kunitz domains. The conserved cysteine residue ShKT domains are labeled by an asterisk; the sequence consensus is indicated by the logo under the alignment.

The protein domains of the putative toxins were annotated by InterProScan. A total of 47 and 55 protein domains were assigned to the *R. esculentum* and *S. malayensis* putative toxins, respectively (Tables S7 and S8). The top ten most represented domains are shown in Figure 6. In general, the results agreed with the BLASTp-ToxClassifier results. Two domains related to hemostasis were found, namely, fibrinogen, alpha/beta/gamma chain, C-terminal globular domain, and coagulation factor 5/8 C-terminal domain, the former being highly represented in both species. Although there were no predominant single protease-related domains, a variety of protease-related domains (peptidase M12B, peptidase M12A, astacin-like metallopeptidase domain, serine proteases/trypsin domain, and disintegrin domain) were found. Furthermore, the domains related to cysteine-rich proteins (CAP domain and SCP domain), the C-type lectin-like domain, and the phospholipase A2 domain were screened in both species. In addition, the ShKT domain and the Kunitz domain, the protein domains with high potential therapeutic value, were also detected in the putative toxins of both species. A total of three and six ShKT domain-containing proteins were identified in the *R. esculentum* and *S. malayensis* putative toxins, respectively, most (seven out of nine) of which were protease-type toxins. In these ShKT domain-containing proteases, the ShKT domain is either associated with the trypsin domain or the peptidase_M12A domain (a metalloproteases domain). Although the sequence of the ShKT domain found in trypsin was slightly different form that of metalloproteases, both of them were characterized by the typical pattern of six cysteines, except the second ShKT domain of Sma_022066-T1 (Sma_022066-T1_M12_ShKT_2), which lacked the fifth cysteine residue of the motif (Figure 7A,B). Furthermore, a total of five Kunitz domains were detected in four protease inhibitor-type putative toxins, with Sma_015170-T1 containing two domains. Most of the detected Kunitz domains displayed the conserved motif of C-8X-C-15X-C-4X-YGGC-12X-C-3X-C. The only exception was the Kunitz domains of Sma_021821-T1, which deviated from the conserved architecture by a Y31F substitution (Figure 7C).

## 3. Discussion

Animal venoms comprise a mixture of bioactive molecules that include different types of toxic proteins. Most of these proteinaceous toxins arise from gene duplication and contain a non-toxic physiological function [19,20]. Owing to this nature of proteinaceous toxins, it is difficult to distinguish between toxic proteins and their non-toxic homologs using BLAST alone. Therefore, most studies based on identifying toxic proteins apply different manual filters to filter out the non-toxic homologs, which may lead to problems in verifying the results. In this study, the protein sequences obtained from proteomic analysis were compared against the UniProt animal venom proteins and toxins database (Tox-Prot) using BLASTp. After that, ToxClassifier, a machine learning model, was used to differentiate toxins from other proteins having non-toxic physiological functions [18]. By using this approach, 190 and 186 putative toxins were predicted from *R. esculentum* and *S. malayensis* transcriptomic data, respectively (Table 1). Furthermore, 40 *R. esculentum* and 51 *S. malayensis* putative toxins were identified at the protein level (Table 2).

Hemostasis-impairing toxins were the most predominant toxin family of both the *R. esculentum* and *S. malayensis* venom proteomes. Most of the toxins in this family share sequence similarity with ryncolins, a group of hemostasis-impairing toxins originally described from *C. rynchops* [21]. Ryncolin genes [22], transcripts, and proteins [23,24,25,26] have also been found in other species of jellyfish, but their functions are not well-characterized. C-type lectins, another major group of hemostasis-impairing toxins, are commonly found in the venoms of a wide variety of animals [27,28,29,30], including several jellyfish species: Pacific sea nettle *Chrysaora fuscescens* [31], Lion’s mane jellyfish *Cyanea capillata,* Nomura’s jellyfish *N. nomurai* [26], and cannonball jellyfish *Stomolophus meleagris* [23]. C-type lectins are calcium-dependent carbohydrate-binding proteins that exhibit pro/anticoagulant, and pro/antithrombotic activities. This type of toxin is also involved in pain and itch sensitization through Toll-like receptors [32]. Therefore, the C-type lectins in *R. esculentum* and *S. malayensis* venom might explain the pruritus and pain caused by the sting.

The prediction of eggNOG-mapper revealed that protease was the most abundant enzyme in the venoms of both species (Figure 5). Consistent with the prediction, 11 proteases were identified as putative toxins in both the *R. esculentum* and *S. malayensis* venoms, which comprise the second most abundant class of toxins. In the venoms of both species, metalloproteases were the predominant proteases. Mainly, they were homologous to zinc metalloproteinase-disintegrin-like and astacin-like metalloprotease, which were also identified in the proteome of the venom of other cnidarians, including Lion’s mane jellyfish *C. capillata* [26], sea wasp *Chironex fleckeri* [33], Pacific sea nettle *C. fuscescens* [31], ghost jellyfish *Cyanea nozakii* [34], *Cyanea sp.* [25], Nomura’s jellyfish *N. nomurai* [24,26], cannonball jellyfish *S. Meleagris* [23], and starlet sea anemone *Nematostella vectensis* [35]. It has been suggested that metalloprotease induces protease-mediated tissue damage by the degradation of the extracellular matrix, which eventually results in necrosis, edema, and hemorrhage [36]. Correspondingly, the skin and tissue necrosis caused by *S. malayensis* envenomation [9] are most likely associated with the highly represented metalloproteases in its venom. In addition, our results are also in agreement with another study, which demonstrated significant metalloprotease activity in *R. esculentum* venom [36]. 

Other than metalloproteases, several serine proteases and serine carboxypeptidases were also identified in the venom proteomes. The presence of proteases has also been reported in the venom of several jellyfish species [24,25,26,31,33]. The functional role of proteases in jellyfish venom is not well understood; while based on observations in other venomous animals, we suggest that the proteases in the venom may be responsible for promoting the spreading and activation of other toxins [37,38]. 

In addition to these two predominated toxin families, other toxins were also identified. Phospholipases, for example, were identified in the venom proteomes of both species, all of which were phospholipases A2 (PLA2s) with two and four copies of PLA2s in *R. esculentum* and *S. malayensis* venom, respectively. PLA2s has also been found in the venom of various other jellyfish species [23,24,25,26,31,33,34,39,40], and the PLA2 activity has been detected in the oral arm of *R. esculentum* [41]. PLA2s are considered hemolysin in jellyfish venom [42]; thus, we suspect that the presence of PLA2s in *R. esculentum* venom might be associated with its hemolytic activities reported previously [43]. Meanwhile, more experiments are required to investigate whether *S. malayensis* also exhibits PLA2-induced hemolytic activity. In addition, the homologs of reticulocalbin, the mediators of PLA2 toxins [44,45], were detected in the venom of both species. This finding implies that PLA2s play an important role in *R. esculentum* and *S. malayensis* envenomation.

Proteases inhibitors, another group of toxins commonly found in jellyfish venom [24,25,26,31,34,46], were also found in the proteomes of *R. esculentum* and *S. malayensis* venom. In our proteomic study, four Kunitz-type serine protease inhibitors were found, two in *R. esculentum* and another two in *S. malayensis* venom, characterized by the conserved motif of C-8X-C-15X-C-4X-YGGC-12X-C-3X-C (Figure 7C). Kunitz-type serine protease inhibitors inhibit proteases, which cause inflammation and interferes with blood coagulation [47]. It has also been suggested that the protease inhibitors also play an auxiliary role in envenomation by maintaining the integrity of the proteinaceous toxins [42]. Moreover, in sea anemones and scorpions, Kunitz-type serine protease inhibitors exhibit an ion channel-blocking function, which might result in paralysis [47]. Further investigation is required to clarify the functions of the protease inhibitors in jellyfish venom.

Other toxins rarely reported in jellyfish venom were also identified in this study. A venom acid phosphatase was identified in the *S. malayensis* venom proteome. This type of toxin is commonly found in honeybees and weever fish, which can induce allergic reaction through the induction of histamine release [48]. To the best of our knowledge, the venom acid phosphatase protein has not been identified in jellyfish venom proteome before. Furthermore, some lethally pore-forming toxins were found in the venom of both species. Three stonustoxins (SNTXs) were identified: One SNTX subunit beta homolog (a hemolysin from estuarine stonefish) and two neoverrucotoxin subunit beta homologs (a hemolysin from reef stonefish) detected in *R. esculentum* and *S. malayensis* venom, respectively. SNTXs show hemolytic and hypotensive activity in stonefish venom [49,50]; however, the function of these toxins in jellyfish venom remains to be tested.

In terms of the discovery of novel therapeutic compounds, our data suggest that *R. esculentum* and *S. malayensis* venoms might be a potential source for drug screening. Our InterProScan results have identified two protein domains with potential therapeutic value, namely, the ShKT domain and the Kunitz domain. The ShKT domain is one of the best-studied protein domains with high therapeutic potential. ShKT is a potent potassium ion channel blocker with high affinity for KV1.3 channels [51]; this channel is essential for effector memory T (TEM) cell activation, which is a hallmark of autoimmune diseases [52,53]. Therefore, KV1.3 is considered a promising target of autoimmune disease treatment. Several modified ShKT peptides with increased selectivity of KV1.3 have been synthetized as potential drugs for treating autoimmune diseases [54,55,56,57,58]. Among them, dalazatide has completed clinical phase 1b [52]. Moreover, Kunitz domain-containing proteases inhibitors were also found in the venoms of both species, which could be considered potential sources of therapeutic compounds. A previous study demonstrated that the potassium ion channel blockade function of Kunitz domain-containing peptides grants them the potential application as neuroprotective drugs [59]. In addition, Kunitz domain-containing proteins also show therapeutic potential in the treatment of cancer [60] and hereditary angioedema [61], attributed to their protease inhibition ability.

The putative toxins identified in this study were predicted based on the toxins recorded in the Tox-Prot database. Further investigations will be needed to confirm the toxicities of these putative toxins and to elucidate their role in envenomation. Regardless, this work revealed that *R. esculentum* and *S. malayensis* nematocysts contain various putative toxins with a high diversity of biological functions. Moreover, our study provides valuable information for the screening of novel therapeutic compounds and an enriched jellyfish toxin database.

## 4. Materials and Methods 

### 4.1. Jellyfish Collection

Specimens of *R. esculentum* and *S. malayensis* were wild-caught and obtained from a local supplier in Hong Kong. *S. malayensis* was also provided by the Ocean Park Hong Kong. Medusae of both species were cultured in circulating artificial seawater (salinity 30 ppt) at room temperature at the Chinese University of Hong Kong. Individuals of *R. esculentum* were fed once per week with newly hatched *Artemia* and were starved for at least two days prior to sampling. Individuals of *S. malayensis* were not fed for several days after arrival in the laboratory before sampling.

### 4.2. Sample Preparation for Proteomic Analysis

Nematocysts were isolated according to methods described in previous studies [26,31]. Protein was extracted from the cleaned nematocysts by dissolving in lysis buffer (6 M urea, 2 M thiourea, and 1 mM dithiothreitol (DTT)). After removing the insoluble impurities by centrifugation at 21,000× *g*, the protein samples were then alkylated with 5 mM of iodoacetamide for 30 min in the dark at room temperature and digested in 1/20 sequencing-grade trypsin (Promega) overnight at 37 °C. The digested peptides were fractionated into four fractions with increasing acetonitrile (ACN) concentrations (7.5%, 12.5%, 17.5%, and 50%) using a high-pH reversed-phase fractionation kit (Thermo Fisher Scientific, Waltham, MA, USA). The fractions were dried in a SpeedVac and resuspended in 5% formic acid (*v*/*v*) and 5% ACN (*v*/*v*). Then, 1 µg of peptides was subjected to nano-flow liquid chromatography separation using a Dionex UltiMate 3000 RSLC nano-system. The sample was separated using a 25-cm-long, 75 µm internal diameter C18 column. The peptides were eluted from the column at a constant flow rate of 0.3 µl/min with a linear gradient from 2% to 35% of ACN over 120 min. The eluted peptides were analyzed by an Orbitrap Fusion Lumos Tribrid mass spectrometer (Thermo Fisher Scientific). MS and MS/MS scans were acquired in the Orbitrap with a mass resolution of 60,000 and 15,000, respectively. MS scan range was from 375 to 1500 *m/z* with an automatic gain control (AGC) target 4e5, and the maximum injection time was 50 ms. The AGC target and the maximum injection time for MS/MS were 5e4 and 250 ms, respectively. The higher-energy collisional dissociation (HCD) mode was used as the fragmentation mode with 30% collision energy. The precursor isolation windows were set to 1.6 *m/z*. 

### 4.3. Spectral Searches and Bioinformatics Analysis

Data were analyzed by Proteome Discoverer version 2.3 with SEQUEST as a search engine. The searching parameters were as follows: oxidation of methionine (+15.9949 Da) and carbamidomethylation of cysteine (+57.0215 Da) was set as the dynamic modification; precursor ion mass tolerance, 10 ppm; fragment ion mass tolerance, 0.02 Da. The data were searched against the translated protein sequences from our constructed transcriptome database obtained previously [15]. The protein level false discovery rate was estimated by Percolator at an experimental q-value (exp. q-value) threshold of 0.05. To identify the putative toxins, the proteins sequences were run against the UniProt animal toxin and venom database to identify toxin contents using BLASTp (e-value of <1.0 × 10^−5^) [17]. Then, BLASTp annotation was validated by ToxClassifier to exclude the proteins with non-toxic physiological functions [18]. GO annotations were done by eggNOG-mapper using the default setting [16]. The signal peptides and subcellular localization were predicted by SignalP-5.0 [62] and DeepLoc-1.0 [63], respectively. The protein domains were screened by InterProScan (5.47–82.0) [46].

## Figures and Tables

**Table 1:** Description of the analysis of the protein databases generated from the *R. esculentum* and *S. malayensis* transcriptomes.

	*R. esculentum*	*S. malayensis*
Proteins sequences	18,923	26,914
Proteins sequences annotated with GO terms	8786	9138
GO terms	143,350	153,009
Biological process GO terms	80,786	24,533
Molecular function GO terms	22,970	41,992
Cellular component GO terms	39,612	86,484
Enzymes	4187	4485
Putative toxins	190	186

**Table 2:** Toxin families identified in each jellyfish species.

	*R. esculentum*		*S. malayensis*	
Toxin Family	UniProt Accession	Description	UniProt Accession	Description
**Hemostasis-impairing toxins**				
	A7X3Z7	C-type lectin lectoxin-Lio2 (CTL)	C6JUN9	C-type lectin (CTL)
	Q593B6	Coagulation factor V (cleaved into coagulation factor V heavy chain and coagulation factor V light chain)	Q593B6	Coagulation factor V (cleaved into coagulation factor V heavy chain and coagulation factor V light chain)
	Q66S03	Galactose-specific lectin nattectin (CTL)	Q4QXT9	Coagulation factor X (EC 3.4.21.6) (cleaved into factor X light chain, factor X heavy chain, and activated factor Xa heavy chain)
	D8VNS8	Ryncolin-2	Q66S03	Galactose-specific lectin nattectin (CTL)
	D8VNT0	Ryncolin-4	D8VNS8	Ryncolin-2
	P22030	Snaclec botrocetin subunit beta (platelet coagglutinin)	D8VNT0	Ryncolin-4
	Q7SZN0	Venom prothrombin activator pseutarin-C non-catalytic subunit (PCNS) (vPA) (venom coagulation factor Va-like protein) (cleaved into pseutarin-C non-catalytic subunit heavy chain and pseutarin-C non-catalytic subunit light chain)	Q56EB0	Snaclec bothrojaracin subunit beta (BJC subunit beta)
	A6MFK7	Venom prothrombin activator vestarin-D1 (vPA) (EC 3.4.21.6) (venom coagulation factor Xa-like protease) (cleaved into vestarin-D1 light chain and vestarin-D1 heavy chain)	Q58L90	Venom prothrombin activator omicarin-C non-catalytic subunit (vPA) (venom coagulation factor Va-like protein) (cleaved into omicarin-C non-catalytic subunit heavy chain and omicarin-C non-catalytic subunit light chain)
			Q58L91	Venom prothrombin activator oscutarin-C non-catalytic subunit (vPA) (venom coagulation factor Va-like protein) (cleaved into oscutarin-C non-catalytic subunit heavy chain and oscutarin-C non-catalytic subunit light chain)
			Q7SZN0	Venom prothrombin activator pseutarin-C non-catalytic subunit (PCNS) (vPA) (venom coagulation factor Va-like protein) (cleaved into pseutarin-C non-catalytic subunit heavy chain and pseutarin-C non-catalytic subunit light chain)
			A6MFK8	Venom prothrombin activator vestarin-D2 (vPA) (EC 3.4.21.6) (venom coagulation factor Xa-like protease) (cleaved into vestarin-D2 light chain and vestarin-D2 heavy chain)
**Proteases**				
	C9D7R2	Astacin-like metalloprotease toxin 2 (EC 3.4.24.-) (Loxosceles astacin-like protease 2) (LALP2)	A0FKN6	Astacin-like metalloprotease toxin 1 (EC 3.4.24.-) (Loxosceles astacin-like protease 1) (LALP) (LALP1)
	Q76B45	Blarina toxin (BLTX) (EC 3.4.21.-)	P0DM62	Astacin-like metalloprotease toxin 5 (EC 3.4.24.-) (Loxosceles astacin-like protease 5) (LALP5) (Fragment)
	K7Z9Q9	Nematocyst-expressed protein 6 (NEP-6) (EC 3.4.24.-) (astacin-like metalloprotease toxin)	Q76B45	Blarina toxin (BLTX) (EC 3.4.21.-)
	W4VS99	Neprilysin-1 (EC 3.4.24.-)	K7Z9Q9	Nematocyst-expressed protein 6 (NEP-6) (EC 3.4.24.-) (astacin-like metalloprotease toxin)
	B2D0J4	Venom dipeptidyl peptidase 4 (allergen C) (venom dipeptidyl peptidase IV) (EC 3.4.14.5) (allergen Api m 5)	W4VS99	Neprilysin-1 (EC 3.4.24.-)
	Q7M4I3	Venom protease (EC 3.4.21.-) (allergen Bom p 4)	B2D0J4	Venom dipeptidyl peptidase 4 (allergen C) (venom dipeptidyl peptidase IV) (EC 3.4.14.5) (allergen Api m 5)
	C9WMM5	Venom serine carboxypeptidase (EC 3.4.16.5) (allergen Api m 9)	O73795	Zinc metalloproteinase/disintegrin (cleaved into snake venom metalloproteinase Mt-b (SVMP) (EC 3.4.24.-) and disintegrin)
	J3S830	Zinc metalloproteinase-disintegrin-like 3a (EC 3.4.24.-) (snake venom metalloproteinase) (SVMP)	P20164	Zinc metalloproteinase-disintegrin-like HR1b (EC 3.4.24.-) (snake venom metalloproteinase) (SVMP) (trimerelysin I) (trimerelysin-1) (cleaved into disintegrin-like 1b)
	F8RKW0	Zinc metalloproteinase-disintegrin-like MTP8 (EC 3.4.24.-) (snake venom metalloproteinase) (SVMP)	A8QL59	Zinc metalloproteinase-disintegrin-like NaMP (EC 3.4.24.-) (snake venom metalloproteinase) (SVMP)
	Q2LD49	Zinc metalloproteinase-disintegrin-like TSV-DM (EC 3.4.24.-) (snake venom metalloproteinase) (SVMP)		
**Phospholipase**				
	P80003	Acidic phospholipase A2 PA4 (PLA2) (EC 3.1.1.4) (phosphatidylcholine 2-acylhydrolase) (cleaved into acidic phospholipase A2 PA2)	P80003	Acidic phospholipase A2 PA4 (PLA2) (EC 3.1.1.4) (phosphatidylcholine 2-acylhydrolase) (cleaved into acidic phospholipase A2 PA2)
	P16354	Phospholipase A2 isozymes PA3A/PA3B/PA5 (PLA2) (EC 3.1.1.4) (phosphatidylcholine 2-acylhydrolase)	I7GQA7	Phospholipase A(2) (EC 3.1.1.4) (phosphatidylcholine 2-acylhydrolase)
			P16354	Phospholipase A2 isozymes PA3A/PA3B/PA5 (PLA2) (EC 3.1.1.4) (phosphatidylcholine 2-acylhydrolase)
**Protease inhibitors**				
	B1P1J3	Cystatin-1 (cystatin JZTX-75)	J3RYX9	Cystatin-1
	P0C8W3	Kunitz-type serine protease inhibitor Hg1 (delta-KTx 1.1)	Q6T269	Kunitz-type serine protease inhibitor bitisilin-3 (two-Kunitz protease inhibitor) (fragment)
			W4VSH9	Kunitz-type U19-barytoxin-Tl1a (U19-BATX-Tl1a) (Kunitz-type serine protease inhibitor Kunitz-1)
**Pore-forming toxins**				
	Q91453	Stonustoxin subunit beta (SNTX subunit beta) (DELTA-synanceitoxin-Sh1b) (DELTA-SYTX-Sh1b) (trachynilysin subunit beta) (TLY subunit beta)	A0ZSK4	Neoverrucotoxin subunit beta (NeoVTX subunit beta)
**Cysteine-rich proteins**				
	Q3SB07	Cysteine-rich venom protein pseudechetoxin-like (CRVP)	A6MFK9	Cysteine-rich venom protein (CRVP) (cysteine-rich secretory protein) (CRISP)
	Q8AVA3	Cysteine-rich venom protein pseudecin (CRVP Pdc)	P81656	Venom allergen 5 (antigen 5) (Ag5) (cysteine-rich venom protein) (CRVP) (allergen Pol d 5)
**Neurotoxins**				
	Q25338	Delta-latroinsectotoxin-Lt1a (delta-LIT-Lt1a) (delta-latroinsectotoxin) (delta-LIT)	G0LXV8	Alpha-latrotoxin-Lh1a (Alpha-LTX-Lh1a) (alpha-latrotoxin) (fragment)
**Other toxins**				
	Q8AY75	Calglandulin	Q92035	Acetylcholinesterase (BfAChE) (EC 3.1.1.7)
	A7ISW2	Glutaminyl-peptide cyclotransferase (EC 2.3.2.5) (glutaminyl cyclase) (QC) (glutaminyl-tRNA cyclotransferase)	A7ISW1	Glutaminyl-peptide cyclotransferase (EC 2.3.2.5) (glutaminyl cyclase) (QC) (glutaminyl-tRNA cyclotransferase)
	Q75WF2	Plancitoxin-1 (EC 3.1.22.1) (plancitoxin I) (plan-I) (cleaved into plancitoxin-1 subunit alpha and plancitoxin-1 subunit beta)	J3S820	Hyaluronidase (EC 3.2.1.35) (hyaluronoglucosaminidase) (venom-spreading factor)
	J3S9D9	Reticulocalbin-2 (taipoxin-associated calcium-binding protein 49 homolog)	A8QL51	l-amino-acid oxidase (Bm-LAAO) (LAO) (EC 1.4.3.2)
	M5B4R7	Translationally controlled tumor protein homolog (GTx-TCTP1)	Q75WF2	Plancitoxin-1 (EC 3.1.22.1) (plancitoxin I) (Plan-I) (cleaved into plancitoxin-1 subunit alpha and plancitoxin-1 subunit beta)
	P0DN11	U-actitoxin-Avd3j (U-AITX-Avd3j) (AsKC7)	J3S9D9	Reticulocalbin-2 (taipoxin-associated calcium-binding protein 49 homolog)
			M5B4R7	Translationally controlled tumor protein homolog (GTx-TCTP1)
			Q5BLY5	Venom acid phosphatase Acph-1 (EC 3.1.3.2) (allergen Api m 3)

## Supplementary Materials

The following are available online at https://www.mdpi.com/1660-3397/18/12/655/s1: Table S1. Putative toxins of the *R. esculentum* protein database; Table S2. Putative toxins of the *S. malayensis* protein database; Table S3. Proteomic analysis results of *R. esculentum*; Table S4. Proteomic analysis results of *S. malayensis*; Table S5. Annotation table for the putative toxins identified in the proteomic analysis for *R. esculentum*; Table S6. Annotation table for the putative toxins identified in the proteomic analysis for *S. malayensis*; Table S7. Protein domains annotated by InterProScan for the *R. esculentum* putative toxins detected by proteomic analysis; Table S8. Protein domains annotated by InterProScan for the *S. malayensis* putative toxins detected by proteomic analysis.

## References

[1] Hagadorn J.W., Dott R.H., Damrow D. (2002). Stranded on a Late Cambrian shoreline: Medusae from central Wisconsin. Geology.

[2] Kayal E., Roure B., Philippe H., Collins A.G., Lavrov D.V. (2013). Cnidarian phylogenetic relationships as revealed by mitogenomics. BMC Evol. Biol..

[3] Shostak S. (2005). Cnidaria (Coelenterates). Encyclopedia of Life Sciences.

[4] WoRMS—World Register of Marine Species. http://www.marinespecies.org/aphia.php?p=browser&accepted=1&id[]=2#focus.

[5] Beckmann A., Özbek S. (2012). The Nematocyst: A molecular map of the Cnidarian stinging organelle. Int. J. Dev. Biol..

[6] Santhanam R. (2020). Venomology of Marine Cnidarians. Biology and Ecology of Venomous Marine Cnidarians.

[7] Remigante A., Costa R., Morabito R., LaSpada G., Marino A., Dossena S. (2018). Impact of scyphozoan venoms on human health and current first aid options for stings. Toxins.

[8] Kawahara M., Uye S., Burnett J., Mianzan H. (2006). Stings of edible jellyfish (Rhopilema hispidum, Rhopilema esculentum and Nemopilema nomurai) in Japanese waters. Toxicon.

[9] Fenner P.J. (2005). Venomous jellyfish of the world. S. Pac. Underw. Med. Soc. J..

[10] Balamurugan E., Reddy B.V., Menon V.P. (2010). Antitumor and antioxidant role of Chrysaora quinquecirrha (sea nettle) nematocyst venom peptide against ehrlich ascites carcinoma in Swiss Albino mice. Mol. Cell. Biochem..

[11] Lee H., Bae S.K., Kim M., Pyo M.J., Kim M., Yang S., Won C.K., Yoon W.D., Han C.H., Kang C. (2017). Anticancer Effect of Nemopilema nomurai Jellyfish Venom on HepG2 Cells and a Tumor Xenograft Animal Model. Evid. Based Complement. Altern. Med..

[12] Ayed Y., Sghaier R.M., Laouini D., Bacha H. (2016). Evaluation of anti-proliferative and anti-inflammatory activities of Pelagia noctiluca venom in Lipopolysaccharide/Interferon-γ stimulated RAW264.7 macrophages. Biomed. Pharmacother..

[13] Ayed Y., Dellai A., Mansour H.B., Bacha H., Abid S. (2012). Analgesic and antibutyrylcholinestrasic activities of the venom prepared from the Mediterranean jellyfish Pelagia noctiluca (Forsskal, 1775). Ann. Clin. Microbiol. Antimicrob..

[14] Taxonomy: “Metazoa [33208]” (Keyword: Toxin OR Annotation: (Type: “Tissue Specificity” Venom)) and Reviewed: Yes. https://www.uniprot.org/uniprot/?query=taxonomy%3A%22Metazoa+%5B33208%5D%22+AND+%28keyword%3Atoxin++OR+annotation%3A%28type%3A%22tissue+specificity%22+AND+venom%29%29+AND+reviewed%3Ayes.

[15] Nong W., Cao J., Li Y., Qu Z., Sun J., Swale T., Yip H.Y., Qian P.Y., Qiu J.W., Kwan H.S. (2020). Jellyfish genomes reveal distinct homeobox gene clusters and conservation of small RNA processing. Nat. Commun..

[16] Huerta-Cepas J., Szklarczyk D., Heller D., Hernández-Plaza A., Forslund S.K., Cook H., Mende D.R., Letunic I., Rattei T., Jensen L.J. (2018). eggNOG 5.0: A hierarchical, functionally and phylogenetically annotated orthology resource based on 5090 organisms and 2502 viruses. Nucleic Acids Res..

[17] Jungo F., Bougueleret L., Xenarios I., Poux S. (2012). The UniProtKB/Swiss-Prot Tox-Prot program: A central hub of integrated venom protein data. Toxicon.

[18] Gacesa R., Barlow D.J., Long P.F. (2016). Machine learning can differentiate venom toxins from other proteins having non-toxic physiological functions. PeerJ Comput. Sci..

[19] Negi S.S., Schein C.H., Ladics G.S., Mirsky H., Chang P., Rascle J.B., Kough J., Sterck L., Papineni S., Jez J.M. (2017). Functional classification of protein toxins as a basis for bioinformatic screening. Sci. Rep..

[20] Hargreaves A.D., Swain M.T., Hegarty M.J., Logan D.W., Mulley J.F. (2014). Restriction and recruitment-gene duplication and the origin and evolution of snake venom toxins. Genome Biol. Evol..

[21] Ompraba G., Chapeaurouge A., Doley R., Devi K.R., Padmanaban P., Venkatraman C., Velmurugan D., Lin Q., Kini R.M. (2010). Identification of a novel family of snake venom proteins veficolins from cerberus rynchops using a venom gland transcriptomics and proteomics approach. J. Proteome Res..

[22] Li Y., Gao L., Pan Y., Tian M., Li Y., He C., Dong Y., Sun Y., Zhou Z. (2020). Chromosome-level reference genome of the jellyfish Rhopilema esculentum. GigaScience.

[23] Li R., Yu H., Xue W., Yue Y., Liu S., Xing R., Li P. (2014). Jellyfish venomics and venom gland transcriptomics analysis of Stomolophus meleagris to reveal the toxins associated with sting. J. Proteom..

[24] Choudhary I., Hwang D.H., Lee H., Yoon W.D., Chae J., Han C.H., Yum S., Kang C., Kim E. (2019). Proteomic analysis of novel components of nemopilema nomurai jellyfish venom: Deciphering the mode of action. Toxins.

[25] Liang H., Jiang G., Wang T., Zhang J., Liu W., Xu Z., Zhang J., Xiao L. (2019). An integrated transcriptomic and proteomic analysis reveals toxin arsenal of a novel Antarctic jellyfish Cyanea sp.. J. Proteom..

[26] Wang C., Wang B., Wang B., Wang Q., Liu G., Wang T., He Q., Zhang L. (2019). Unique Diversity of Sting-Related Toxins Based on Transcriptomic and Proteomic Analysis of the Jellyfish Cyanea capillata and Nemopilema nomurai (Cnidaria: Scyphozoa). J. Proteome Res..

[27] Gutiérrez J.M., Sanz L., Escolano J., Fernández J., Lomonte B., Angulo Y., Rucavado A., Warrell D.A., Calvete J.J. (2008). Snake venomics of the lesser antillean pit vipers bothrops caribbaeus and Bothrops lanceolatus: Correlation with toxicological activities and immunoreactivity of a heterologous antivenom. J. Proteome Res..

[28] Öhler M., Georgieva D., Seifert J., VonBergen M., Arni R.K., Genov N., Betzel C. (2010). The venomics of bothrops alternatus is a pool of acidic proteins with predominant hemorrhagic and coagulopathic activities. J. Proteome Res..

[29] Valenzuela J.G., Garfield M., Rowton E.D., Pham V.M. (2004). Identification of the most abundant secreted proteins from the salivary glands of the sand fly Lutzomyia longipalpis, vector of Leishmania chagasi. J. Exp. Biol..

[30] Magalhães G.S., Junqueira-de-Azevedo I.L.M., Lopes-Ferreira M., Lorenzini D.M., Ho P.L., Moura-da-Silva A.M. (2006). Transcriptome analysis of expressed sequence tags from the venom glands of the fish Thalassophryne nattereri. Biochimie.

[31] Ponce D., Brinkman D.L., Potriquet J., Mulvenna J. (2016). Tentacle transcriptome and venom proteome of the pacific sea nettle, Chrysaora fuscescens (Cnidaria: Scyphozoa). Toxins.

[32] Ishikawa A., Miyake Y., Kobayashi K., Murata Y., Iizasa S., Iizasa E., Yamasaki S., Hirakawa N., Hara H., Yoshida H. (2019). Essential roles of C-type lectin Mincle in induction of neuropathic pain in mice. Sci. Rep..

[33] Brinkman D.L., Jia X., Potriquet J., Kumar D., Dash D., Kvaskoff D., Mulvenna J. (2011). Transcriptome and venom proteome of the box jellyfish Chironex fleckeri. BMC Genom..

[34] Li R., Yu H., Yue Y., Liu S., Xing R., Chen X., Li P. (2016). Combined proteomics and transcriptomics identifies sting-related toxins of jellyfish Cyanea nozakii. J. Proteom..

[35] Moran Y., Praher D., Schlesinger A., Ayalon A., Tal Y., Technau U. (2013). Analysis of Soluble Protein Contents from the Nematocysts of a Model Sea Anemone Sheds Light on Venom Evolution. Mar. Biotechnol..

[36] Lee H., Jung E., Kang C., Yoon W.D., Kim J.S., Kim E. (2011). Scyphozoan jellyfish venom metalloproteinases and their role in the cytotoxicity. Toxicon.

[37] Almeida F.M., Pimenta A.M.C., DeFigueiredo S.G., Santoro M.M., Martin-Eauclaire M.F., Diniz C.R., DeLima M.E. (2002). Enzymes with gelatinolytic activity can be found in Tityus bahiensis and Tityus serrulatus venoms. Toxicon.

[38] Serrano S.M.T., Maroun R.C. (2005). Snake venom serine proteinases: Sequence homology vs. substrate specificity, a paradox to be solved. Toxicon.

[39] Li A., Yu H., Li R., Liu S., Xing R., Li P. (2019). Inhibitory Effect of Metalloproteinase Inhibitors on Skin Cell Inflammation Induced by Jellyfish Nemopilema nomurai Nematocyst Venom. Toxins.

[40] Kim H.M., Weber J.A., Lee N., Park S.G., Cho Y.S., Bhak Y., Lee N., Jeon Y., Jeon S., Luria V. (2019). The genome of the giant Nomura’s jellyfish sheds light on the early evolution of active predation. BMC Biol..

[41] Zhu S., Ye M., Xu J., Guo C., Zheng H., Hu J., Chen J., Wang Y., Xu S., Yan X. (2015). Lipid Profile in Different Parts of Edible Jellyfish Rhopilema esculentum. J. Agric. Food Chem..

[42] Liu G., Zhou Y., Liu D., Wang Q., Ruan Z., He Q., Zhang L. (2015). Global transcriptome analysis of the tentacle of the Jellyfish Cyanea capillata using deep sequencing and expressed sequence tags: Insight into the toxin-and degenerative disease-related transcripts. PLoS ONE.

[43] Yu H., Li C., Li R., Xing R., Liu S., Li P. (2007). Factors influencing hemolytic activity of venom from the jellyfish Rhopilema esculentum Kishinouye. Food Chem. Toxicol..

[44] Hseu M.-J., Yen C.-H., Tzeng M.-C. (1999). Crocalbin: A new calcium-binding protein that is also a binding protein for crotoxin, a neurotoxic phospholipase A 2. FEBS Lett..

[45] Dodds D., Schlimgen A.K., Lu S.-Y., Perin M.S. (2002). Novel Reticular Calcium Binding Protein Is Purified on Taipoxin Columns. J. Neurochem..

[46] Jones P., Binns D., Chang H.-Y., Fraser M., Li W., Mcanulla C., Mcwilliam H., Maslen J., Mitchell A., Nuka G. (2014). InterProScan 5: Genome-scale protein function classification. Bioinformatics.

[47] Mourão C.B.F., Schwartz E.F. (2013). Protease inhibitors from marine venomous animals and their counterparts in terrestrial venomous animals. Mar. Drugs.

[48] Gorman L.M., Judge S.J., Fezai M., Jemaà M., Harris J.B., Caldwell G.S. (2020). The venoms of the lesser (Echiichthys vipera) and greater (Trachinus draco) weever fish—A review. Toxicon X.

[49] Sung J.M.L., Low K.S.Y., Khoo H.E. (2002). Characterization of the mechanism underlying stonustoxin-mediated relaxant response in the rat aorta in vitro. Biochem. Pharmacol..

[50] Yew W.S., Khoo H.E. (2000). The role of tryptophan residues in the hemolytic activity of stonustoxin, a lethal factor from stonefish (*Synanceja horrida*) venom. Biochimie.

[51] Pennington M.W., Byrnes M.E., Zaydenberg I., Khaytin I., de Chastonay J., Krafte D.S., Hill R., Mahnir V.M., Volberg W.A., Gorczyca W. (1995). Chemical synthesis and characterization of ShK toxin: A potent potassium channel inhibitor from a sea anemone. Int. J. Pept. Protein Res..

[52] Chandy K.G., Norton R.S. (2017). Peptide blockers of Kv1.3 channels in T cells as therapeutics for autoimmune disease. Curr. Opin. Chem. Biol..

[53] Beeton C., Wulff H., Standifer N.E., Azam P., Mullen K.M., Pennington M.W., Kolski-Andreaco A., Wei E., Grino A., Counts D.R. (2006). Kv1.3 channels are a therapeutic target for T cell-mediated autoimmune diseases. Proc. Natl. Acad. Sci. USA.

[54] Tarcha E.J., Chi V., Muñoz-Elías E.J., Bailey D., Londono L.M., Upadhyay S.K., Norton K., Banks A., Tjong I., Nguyen H. (2012). Durable pharmacological responses from the peptide ShK-186, a specific Kv1.3 channel inhibitor that suppresses T cell mediators of autoimmune disease. J. Pharmacol. Exp. Ther..

[55] Pennington M.W., Chang S.C., Chauhan S., Huq R., Tajhya R.B., Chhabra S., Norton R.S., Beeton C. (2015). Development of highly selective Kv1.3-blocking peptides based on the sea anemone peptide ShK. Mar. Drugs.

[56] Pennington M.W., Harunur Rashid M., Tajhya R.B., Beeton C., Kuyucak S., Norton R.S. (2012). A C-terminally amidated analogue of ShK is a potent and selective blocker of the voltage-gated potassium channel Kv1.3. FEBS Lett..

[57] Chang S.C., Huq R., Chhabra S., Beeton C., Pennington M.W., Smith B.J., Norton R.S. (2015). N-terminally extended analogues of the K+ channel toxin from Stichodactyla helianthus as potent and selective blockers of the voltage-gated potassium channel Kv1.3. FEBS J..

[58] Pennington M.W., Beeton C., Galea C.A., Smith B.J., Chi V., Monaghan K.P., Garcia A., Rangaraju S., Giuffrida A., Plank D. (2009). Engineering a stable and selective peptide blocker of the Kv1.3 channel in T lymphocytes. Mol. Pharmacol..

[59] Liao Q., Li S., Siu S.W.I., Yang B., Huang C., Chan J.Y.W., Morlighem J.É.R.L., Wong C.T.T., Rádis-Baptista G., Lee S.M.Y. (2018). Novel Kunitz-like Peptides Discovered in the Zoanthid Palythoa caribaeorum through Transcriptome Sequencing. J. Proteome Res..

[60] Ranasinghe S.L., Rivera V., Boyle G.M., McManus D.P. (2019). Kunitz type protease inhibitor from the canine tapeworm as a potential therapeutic for melanoma. Sci. Rep..

[61] Farkas H., Varga L. (2011). Ecallantide is a novel treatment for attacks of hereditary angioedema due to C1 inhibitor deficiency. Clin. Cosmet. Investig. Dermatol..

[62] Almagro Armenteros J.J., Tsirigos K.D., Sønderby C.K., Petersen T.N., Winther O., Brunak S., von Heijne G., Nielsen H. (2019). SignalP 5.0 improves signal peptide predictions using deep neural networks. Nat. Biotechnol..

[63] Almagro Armenteros J.J., Sønderby C.K., Sønderby S.K., Nielsen H., Winther O. (2017). DeepLoc: Prediction of protein subcellular localization using deep learning. Bioinformatics.

